# Outcomes of Medial Open Wedge High Tibial Osteotomy in a University Hospital

**DOI:** 10.5704/MOJ.2603.003

**Published:** 2026-03

**Authors:** MH Nasuruddin, AA Abbas, AM Merican, KA Ayob, MS Hashim, V Selvaratnam

**Affiliations:** 1 Department of Orthopaedic, Traumatology, and Rehabilitation, International Islamic University Malaysia, Kuantan, Malaysia; 2 National Orthopaedic Centre of Excellence for Research and Learning (NOCERAL), Department of Orthopaedic Surgery, Universiti Malaya, Kuala Lumpur, Malaysia

**Keywords:** knee osteoarthritis, medial open wedge high tibial osteotomy, unicompartmental knee osteoarthritis, Malaysian patients, knee injury and osteoarthritis outcome score

## Abstract

**Introduction:**

Medial open wedge high tibial osteotomy (MOWHTO) is one of the modalities to treat unicompartmental knee osteoarthritis (KO). Many studies have shown good outcomes of MOWHTO, but there are no published series of Malaysian patients. The aim of this study is to determine the outcome of MOWHTO in primary medial compartment KO in a university teaching hospital in Malaysia.

**Materials and methods:**

This is a retrospective study of patients who underwent MOWHTO in our Joint Reconstruction Unit between 2017 and 2022 with a minimum of 12 months follow-up.

**Results:**

Data from a total of 15 knees were reviewed. The mean age of patients was 41.8 years, and the mean BMI was 31.7. The mean Kellgren-Lawrence (KL) osteoarthritis grade was 2.87 (±0.52). The mean hip knee angle (HKA) pre-operatively was 12.26° varus and post-operatively was 3.33° valgus. Mean correction of HKA was 13.59°. The results revealed an improvement in all patients, as observed from the significant mean difference between pre-operative (38.53) and post-operative (77.60) Knee Injury and Osteoarthritis Outcome (KOOS) (p-value<0.001) scores. Patients who were above 50 years old showed a significantly better improvement in KOOS score compared to those below 50 (p-value <0.05). There was no significant difference observed between BMI and KOOS score improvement (F-stat=0.580, p-value >0.05).

**Conclusion:**

MOWHTO is a good treatment option in medial compartment primary KO with varus deformity among Malaysian population. A larger sample size with a longer follow-up period is needed to draw a definitive conclusion.

## Introduction

Knee osteoarthritis (KO) is a degenerative joint disease affecting the knee joint. It is one of the major global health issues, commonly occurring in ageing individuals. According to the Global Burden of Disease study in 2019, the prevalence of KO indicates an increasing trend globally, with a 113.25 percent increase from 1999 to 20191. Similarly, in Malaysia, KO is the most common form of osteoarthritis with increasing prevalence over the previous decades^2,3^.

In unicompartmental KO, the symptoms only occur in one compartment of the knee joint. It is characterised by the abnormality at the articular cartilage in the medial or lateral compartment of the joint^4^. Patients with isolated medial compartment KO experience pain in the medial compartment of the knee during daily activities and sometimes even at rest.

Treatment of KO depends on the severity of the disease, level of pain, physical limitation, and medical history of the individuals. Moreover, the conservative treatment care for KO is costly and economically burdensome. In the United States the cost ranges between USD 4000 to USD 30000 per person annually^5^. Surgical management is offered in cases which has failed non-operative options. High tibial osteotomy (HTO) is one of the established operative options for treating unicompartmental KO^6,7^. Other surgical management options are unicompartmental knee arthroplasty (UKA) and total knee arthroplasty (TKA)^8^. Medial open wedge high tibial osteotomy (MOWHTO) involves realigning the tibia to relieve pressure on the diseased medial knee compartment.

Despite many studies showing good outcomes of HTO, to the authors’ knowledge, there are no published series specifically looking at the Malaysian population with medial unicompartmental KO. This procedure is still not widely offered as a surgical option by orthopaedic surgeons in Malaysia to treat this condition^3,9^. With the continuous and advancing improvement in joint preserving surgeries, the assessment of the outcomes of MOWHTO as one of the treatment modalities of KO requires further investigation.

The aims of this study are to evaluate the outcomes of MOWHTO in KO patients performed in our centre and to identify the associated factors influencing the outcome of this surgical treatment. Specifically, this study seeks to address the following research questions: (1) to examine the correlation between pre-operative and post-operative KOOS score; and (2) to identify whether there are significant differences between the age at surgery, BMI, and severity score in terms of pre-operative and post-operative KOOS score.

## Materials And Methods

This is a retrospective, single institution study. All patients who were diagnosed with medial compartment osteoarthritis who had undergone MOWHTO between 2017 and 2022 with a minimum of 12 months follow-up were included in this study. We identified 16 patients who had a MOWHTO for medial compartment OA. One patient was excluded from the study as she had a double-level osteotomy. Therefore, 15 patients were included in the study. No patients had any underlying bone metabolic diseases. However, data regarding other co-morbidities were not available. Data collected included demographic data, Kellgren Lawrence (KL) classification, Hip Knee Angle (HKA) correction, body mass index (BMI), pre- and post-operative Knee Injury and Osteoarthritis Outcome Score (KOOS).

**Fig. 1:**
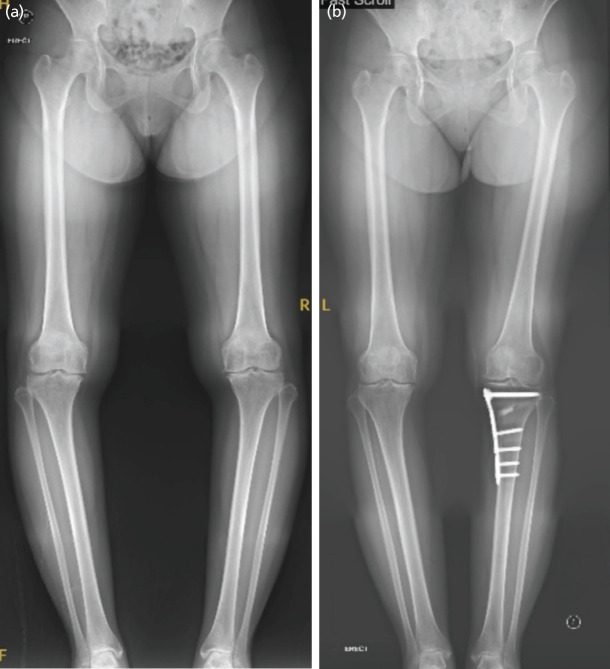
(a) Pre-operative and (b) one-year post-operative left MOWHTO long leg alignment radiographs with a Tomofix plate and Chronos.

**Fig. 2:**
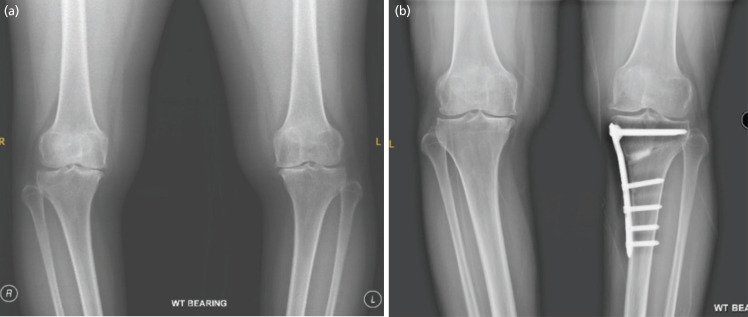
(a) Pre-operative and (b) one-year post-operative left MOWHTO AP Knee radiograph showing a united osteotomy and opening up of medial joint space.

All surgical procedures were performed by three surgeons. All HTO were fixed with a medial high tibial locking plate [Tomofix® Osteotomy System] and the gap was filled with bone substitute [chronOS® Bone Graft Substitute] (Fig. 1 and Fig. 2). All patients had a knee arthroscopy prior to their MOWHTO in the same sitting to make sure there was no significant osteoarthritis changes in the lateral compartment. Patient with degenerative medial meniscal tear had a partial medial meniscectomy and chondroplasty was performed for chondral injury. However detailed data on arthroscopic procedures were not retrospectively available. No other procedures, such as microfracture, were performed. Postoperatively, patients were allowed partial weight bearing as tolerated with crutches for six weeks after showing evidence of union. There was no incidence of delayed union or nonunion. A hinge knee brace was used for the first six weeks locked from 0° – 90°. Full weight bearing and full range of movement was started after six weeks without a brace. All patients underwent physiotherapy throughout the postoperative period for strengthening and range of movement exercises.

The collected data were analysed using SPSS. An alpha of 0.05 was used for all tests. The descriptive measures were used to quantify the distribution of the patients by their demographic and treatment scores. Correlation analyses were done to examine the correlations between age at surgery, Kellgren Lawrence (KL) classification, and Hip Knee Angle correction (HKA) with KOOS scores of the study subjects. Independent t-tests was used to compare means of: (1) pre-operative and post-operative KOOS score; (2) KOOS score by different age at surgery; and (3) KOOS score by BMI.

## Results

In terms of clinical background, descriptive results in Table I explains the distribution of data collected for a total of 15 study subjects. In terms of age, majority of the patients were below 50 years when they had their MOWHTO (73.3%; n=11), while only four (26.7%) were above 50 years of age. The mean value of KL classification was 2.87 (±0.52). The mean BMI value was 31.73 (±7.04), whereby majority of the patients were categorised as obese (73.3%; n=11), followed by overweight (20%; n=3) and underweight individuals (6.7%; n=1). The mean of HKA correction was 13.59° (±4.02), whereby the mean pre-op HKA was 10.26° varus (±3.47) and post-op HKA was 3.33° valgus (±2.69).

**Table I:** Clinical background of MOWHTO patients.

	Frequency, n (%)
Age at surgery (Mean±SD*)	41.80 ± 11.25
Age at surgery	
< 50 years	11 (73.3)
> 50 years	4 (26.7)
Kellgren-Lawrence (KL) classification (Mean±SD*)	2.87±0.52
BMI (Mean±SD*)	31.73±7.04
BMI Category	
Underweight	1 (6.7%)
Normal	0 (0%)
Overweight	3 (20%)
Obese	11 (73.3%)
Total Hip-Knee-Angle (HKA) correction (Mean±SD*)	
Pre-opeative (varus)	10.26° ± 3.47
Post-operative (valgus)	3.33° ± 2.69
Correction	13.59° ± 4.02

Next, paired sample and independent t-test were applied to compare the outcomes within and between groups. Table II shows the results of paired sample t-test for comparing the mean differences of pre-operative and post-operative KOOS score. The p-value revealed a significant mean difference between pre-op and post-op scores. The post-operative score was 77.60 which was significantly higher and at least twice than the mean pre-op score (38.53). This result suggests that there was a significant improvement among the MOWHTO patients.

**Table II:** KOOS (pre-operative vs post-operative score) mean difference.

	Mean	Std. Deviation	p-value*
Pre score	38.53	15.94	<0.001
Post score	77.60	14.37	

Note - *Paired t-test

An independent t-test was done to compare means of KOOS by different age-at-surgery groups. As can be observed from the results in Table III, there was a statistically significant mean difference KOOS score between patients who were older and those less than 50 years old (p<0.05). Specifically, older patients (i.e. more than 50 years) had a significantly higher mean improvement of KOOS score (53.75; ±11.32) compared to those below 50 years (33.72; ±14.60). This indicates that the outcome of MOWHTO varied significantly by their age at surgery whereby the older group showed better post-operative improvement compared to those below 50 years of age.

**Table III:** Mean comparison of KOOS by different age-at-surgery groups.

	Age at surgery	Mean	Std. Deviation	t-stat	p-value*
Knee Injury and Osteoarthritis	< 50 years	33.72	14.60	-2.47	0.028
Outcome Score (KOOS)	> 50 years	53.75	11.32		

Note - *Independent t-test

One-Way ANOVA test was used to compare the means of KOOS of different BMI categories, and the results are summarised in Table IV. As indicated by the p-value, no statistically significant mean difference was observed in the KOOS among the four BMI groups in this study (F-stat=0.580, p>0.05). Thus, the patients’ MOWHTO outcome did not vary significantly by their BMI.

**Table IV:** Mean comparison of KOOS by BMI.

	BMI	Mean	Std. Deviation	F-stat	p-value*
Knee Injury and Osteoarthritis	Underweight (<18.5)	43.00	0	0.580	0.576
Outcome Score (KOOS)	Normal (18.5–22.9)	0	0		
	Overweight (23-24.9)	36.45	16.26		
	Obese (>25)	38.93	17.53		

Note - *One-Way ANOVA test

Finally, correlation analyses were run to identify whether there were significant relationships between the study variables, i.e., age at surgery, KL classification, and KOOS, as shown in Table V. No significant correlations were found in both relationship (p>0.05). Based on the correlation coefficient (r), the correlation between the patients’ age at surgery and KOOS was weak and not significant (26.2%; p>0.05). Additionally, there was a negative and very weak correlation between KL classification and KOOS, which was similarly not significant (-1.6%; p>0.05). Therefore, MOWHTO outcomes were not correlated to their age and the severity level of the disease. No patients developed postoperative complications or needed further additional surgeries.

**Table V:** Correlations between age at surgery, KL classification, and KOOS.

		Knee Injury and Osteoarthritis Outcome Score (KOOS)*
Age at surgery	Pearson Correlation	26.2%
	Sig. (2-tailed)	0.346
	N	15
KL classification	Pearson Correlation	-1.6%
	Sig. (2-tailed)	0.955
	N	15

Note - *Pearson Correlation

## Discussion

This study reviewed the medical records of 15 KO patients with a minimum of 12 months follow-up after their MOWHTO over the last 6 years in a university teaching hospital in Malaysia. KOOS score was used as a patient-reported outcome measurement to assess both short-term and mid-term surgical outcomes from the patients' perspective, as it has been shown that it is a good indicator of changes in symptoms of KO patients^10,11^. The most important finding obtained from this study is the statistically significant improvement in patients' treatment outcomes based on a higher post-operative KOOS score compared to their preoperative score, which potentially suggests the effectiveness of this surgical procedure in treating unicompartmental KO.

This finding has highlighted the potential of MOWHTO as an effective surgical procedure to correct deformities and symptoms related to KO. Various studies in the literature have shown good outcomes of MOWHTO in treating medial unicompartmental KO. For instance, medium- and long-term efficiency of this treatment in reducing and eliminating patients' KO severity has been reported^12^. In other studies, significant improvements after MOWHTO treatment were observed in the clinical scores of 65 knees^13^ and KOOS scores of 85 knees^14^ after 1 to 3 years and 3 to 7 years follow-up, respectively.

This present study also highlighted the effect of age on patient-related outcomes after MOWHTO. Specifically, statistically significant improvement by the age-at-surgery was observed in patients after MOWHTO based on the mean differences of the scores, whereby patients above 50 years old had significantly higher mean KOOS score compared to those below 50. This contrasts with some recent studies that reported no significant difference in terms of age in patients who had MOWHTO for KO^10,15,16^. Skogö Nyvang *et al*^17^ observed a significant age difference, although younger patients have significantly greater affective pain expression and thus greater emotional impact compared to the elderly. Song *et al* found that cartilage status, rather than chronologic age, determines the outcomes of open wedge high tibial osteotomy^18^.

On the contrary, this study did not observe any significant difference in KOOS scores by their BMI categories. This somehow suggests that there is a similar degree of perceived clinical outcomes and health-related quality of life after an MOWHTO among KO patients regardless of their BMI. Similarly reported by Herbst *et al*^19^, overweight patients may receive the same extent of benefits from an MOWHTO with similar complication rates as those with a normal BMI. In another study, TKA patients under the obese BMI category reported similar benefits to those in the normal weight category^20^. This is also similar with the study by Overgaard *et al*^21^ which found that patient-reported one-year outcome of knee arthroplasty patients was not affected by BMI. Despite this, contradicting evidence was observed in another study^22^ whereby BMI or level of obesity was reported to be one of the associating factors with the OA patients clinical and functional outcomes.

This study has a notable limitation regarding the distribution of BMI categories among participants, as the majority of patients were categorised as overweight or obese, with only a single patient classified as underweight and none in the normal BMI range. This imbalanced representation limits the generalisability of our BMI-related findings. Future studies should aim for a more balanced patient cohort in terms of BMI categories or apply statistical adjustments to better elucidate the influence of BMI on the outcomes of MOWHTO.

Another limitation is the small number of patients and relatively short follow-up period. It is difficult to draw definitive conclusions with a small sample size. Most patients in our population tend to present late when they have KO. They only opt for surgery when their KO is at the end stage with severe deformity when MOWHTO is no longer an option. A longer follow-up period with a large sample size is necessary to draw a definitive conclusion regarding the effectiveness of MOWHTO. Moreover, this study also did not include a control group with an alternative treatment for unicompartmental KO. It would be beneficial for future research to analyse such comparison to confirm the effectiveness of MOWHTO.

Moreover, although all patients underwent diagnostic arthroscopy prior to MOWHTO, detailed data on the specific arthroscopic procedures performed (e.g., partial meniscectomy, chondroplasty) were not captured. The lack of documentation prevents subgroup analysis and may confound the interpretation of surgical outcomes.

Finally, results obtained in this study should also be considered in terms of variability of surgical techniques due to the involvement of multiple surgeons. The findings are also limited in terms of the dimensions covered in the research instrument. As suggested by Kuwashima *et al*^10^, other data collection tools like KSS includes the dimensions of patients’ satisfaction and expectation which are not covered in KOOS score. Therefore, this study can be replicated by future research by focusing on other outcome dimensions of HTO.

Findings obtained in this study has provided several important practical implications. The first important recommendation is the role of MOWHTO in treating unicompartmental KO. Next, age-specific interventions can be employed in the post-operative management of KO patients that suits their age-related needs and ability in order to ensure their optimal recovery and prevent further development of chronic pain and complications. Finally, regarding the role of BMI, this study has put forward the practical evaluation of patient-reported knee treatment outcomes, regardless of their body-weight conditions. Although BMI was found to be insignificantly correlated to the patients’ perceived outcome in terms of KOOS score, obesity should still be considered as one of the primary risk factors for KO. This is considering the fact that the number of individuals affected by OA is expected to increase to one in five by 2031, with the increasing rate of ageing population and rates of obesity^23^.

## Conclusion

In summary, this study shows good outcomes of MOWHTO among KO patients in our centre, thus suggesting that it is a good treatment option for medial compartment primary KO with varus deformity among Malaysian population. Further research with a longer follow-up period and large sample size is necessary to draw a definitive conclusion.
